# Beyond Glycemic Control in Diabetes Mellitus: Effects of Incretin-Based Therapies on Bone Metabolism

**DOI:** 10.3389/fendo.2013.00073

**Published:** 2013-06-18

**Authors:** Elena Ceccarelli, Elisa G. Guarino, Daniela Merlotti, Aurora Patti, Luigi Gennari, Ranuccio Nuti, Francesco Dotta

**Affiliations:** ^1^Diabetes Unit, Department of Medicine, Surgery and Neuroscience, University of Siena, Siena, Italy; ^*2*^Internal Medicine Unit, Department of Medicine, Surgery and Neuroscience, University of Siena, Siena, Italy; ^*3*^Fondazione Umberto Di Mario ONLUS, Siena, Italy

**Keywords:** type 2 diabetes, incretin hormones, GLP-1, incretin-based therapy, bone metabolism, osteoporosis

## Abstract

Diabetes mellitus (DM) and osteoporosis (OP) are common disorders with a significant health burden, and an increase in fracture risk has been described both in type 1 (T1DM) and in type 2 (T2DM) diabetes. The pathogenic mechanisms of impaired skeletal strength in diabetes remain to be clarified in details and they are only in part reflected by a variation in bone mineral density. In T2DM, the occurrence of low bone turnover together with a decreased osteoblast activity and compromised bone quality has been shown. Of note, some antidiabetic drugs (e.g., thiazolidinediones, insulin) may deeply affect bone metabolism. In addition, the recently introduced class of incretin-based drugs (i.e., GLP-1 receptor agonists and DPP-4 inhibitors) is expected to exert potentially beneficial effects on bone health, possibly due to a bone anabolic activity of GLP-1, that can be either direct or indirect through the involvement of thyroid C cells. Here we will review the established as well as the putative effects of incretin hormones and of incretin-based drugs on bone metabolism, both in preclinical models and in man, taking into account that such therapeutic strategy may be effective not only to achieve a good glycemic control, but also to improve bone health in diabetic patients.

## Introduction

Type 2 diabetes mellitus (T2DM) is characterized by insulin resistance and by a progressive functional loss of pancreatic beta cells. Islet dysfunction is characterized by failure of beta cells to compensate for insulin resistance and by a concomitant enhanced glucagon release from alpha cells. Of note, an alteration of insulin/glucagon balance seems to be one of the key factors in disease physiopathology (Butler et al., 2003; Giorgino et al., 2005; Marchetti et al., 2008). An appropriate strategic approach for T2DM treatment should therefore target insulin resistance, beta-cell dysfunction, and increased glucagon levels.

The role of incretin hormones (e.g., GLP-1, glucagon like peptide 1; GIP, glucose-dependent insulinotropic peptide) in T2DM therapy has recently received much attention, because of the beneficial actions of these molecules on the pancreatic islet (Ahren, 2003; Marchetti et al., 2012). Incretin-based therapy encompasses two classes of drugs: GLP-1 receptor agonists and dipeptidyl peptidase-4 (DPP-4) inhibitors. Of note, in the light of physiological actions of incretin hormones, such drugs are expected to exert potentially beneficial effects beyond glycemia, including on bone health, possibly due to a bone anabolic activity of GLP-1 that can be either direct or indirect, through the involvement of thyroid C cells. This is of potential interest, in the light that an increase in fracture risk has been described both in type 1 diabetes (T1DM) and in T2DM (Hamann et al., 2012). The pathogenic mechanisms of impaired skeletal strength in diabetes remain to be clarified in detail, and they are only in part reflected by a variation in bone mineral density (BMD). In T2DM, the occurrence of low bone turnover has been shown together with decreased osteoblast activity and compromised bone quality. Of note, some antidiabetic drugs (e.g., thiazolidinediones, insulin) may have important effects on bone metabolism.

Here we review the established as well as the putative effects of incretin-based drugs on bone metabolism, both in preclinical models and in man, taking into account that such therapeutic strategy may be effective not only to achieve a good glycemic control, but also to improve bone health in diabetic patients.

A literature search was performed screening PubMed database using combinations of the following search terms: “DM”; “type 1 diabetes”; “type 2 diabetes”; “bone metabolism”; “incretin hormones”; “GLP-1”; “incretin-based therapy”; “DPP-4 inhibitors.” This initial screening identified a total of 2492 records. We then included only publications published in English, removed duplicates, and took into final consideration those articles directly examining the relationship between incretin hormones or incretin-based therapies with bone metabolism and bone turnover markers in diabetic patients, in experimental animal models and in cell lines. Overall, 110 publications were included in our review.

## Incretin Hormones and Incretin-Based Therapies: Effects Beyond Glycemia

**Figure 1:**
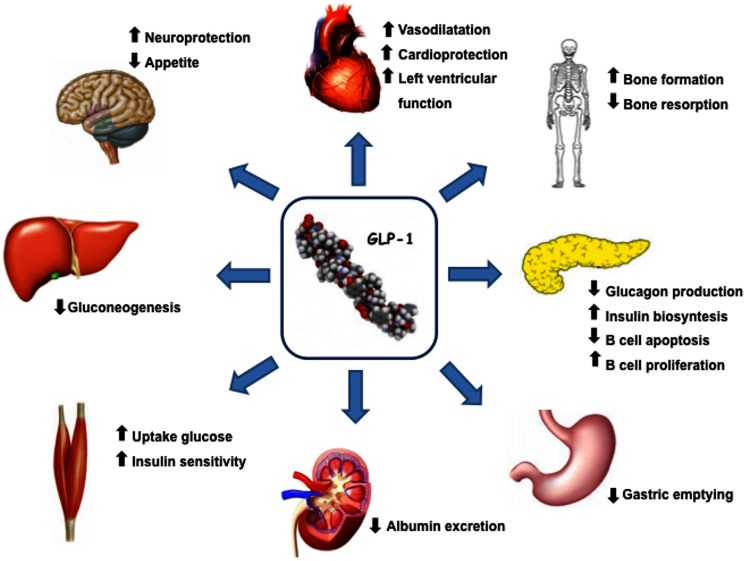
**GLP-1 actions and target organs**. GLP-1, through its receptor GLP-1R, has functional effects on a variety of tissues.

Glucagon like peptide 1(7–36) is produced by intestinal L-cells in response to meal and is a pluripotent incretin hormone in humans, which exerts multiple physiological actions (Mojsov et al., 1987). GLP-1(7–36) is promptly cleaved by the enzyme dipeptidyl peptidase-4 (DPP-4) into its inactive form GLP-1(9–36) (Lovshin and Drucker, 2009). GLP-1 receptors (GLP-1R) are widely expressed in pancreatic islet cells and in several other tissues. GLP-1 has a wide target tissue distribution and acts through specific heterotrimeric G-protein complex receptors, functionally associated with activation of second messengers such as adenylate cyclase. GLP-1R belongs to the class B family of 7-transmembrane-spanning receptors and its expression has been reported in pancreas, heart, vascular smooth muscle cells, endothelial cells, macrophages and monocytes, lung, kidney, gastrointestinal tract (stomach and intestine), as well as in central (brain) and in peripheral nervous system. Brain and cardiac tissues express the same GLP-1R as pancreas, whereas receptors on skeletal muscle, adipose tissue, and liver bear some degree of homology to pancreatic GLP-1R (Rasmussen et al., 2003; Holst and Jens, 2008; Martin et al., 2011). GLP-1, through its receptor GLP-1R, has multiple physiological actions (Figure 1), including enhancement of glucose-stimulated insulin secretion by pancreatic β-cells, inhibition of glucose-dependent glucagon secretion, and control of appetite and body weight (Kieffer and Habener, 1999).

### Incretin hormones and diabetic vascular complications

In the light of above-mentioned properties, new drugs based on incretin effects have been and are being developed for T2DM treatment. GLP-1R-agonists exert multiple effects on cardiovascular system, such as modulation of blood pressure, of heart rate, of arterial dilation, of endothelial and myocardial function, and of myocardial contractility (Vila Petroff et al., 2001; Yamamoto et al., 2002; Green et al., 2008). Beneficial effects of GLP-1 on cardiovascular diseases have been reported both in animal models and in patients, either in the absence or presence of diabetes (Nikolaidis et al., 2005a,b; Ozyazgan et al., 2005; Sokos et al., 2006). Binding of GLP-1 to its receptor in the myocardium leads to an increased production of cyclic adenosine monophosphate (cAMP) and to an activation of protein kinase A (PKA), which results into an increased glucose uptake and into inotropic effects in myocardial tissue. GLP-1 knockout mice exhibit reduced resting heart rate, elevated left ventricular and diastolic pressure, and increased left ventricular thickness compared to wild type mice (Ban et al., 2008). Treatment with GLP-1 or with GLP-1R-agonists was found to improve left ventricular function (Nikolaidis et al., 2004; Sokos et al., 2006) and to reduce circulating levels of brain natriuretic peptide (BNP) (Courrèges et al., 2008; Bergenstal et al., 2010).

Elevated tumor necrosis factor-alpha (TNF-α) levels and hyperglycemia are involved in diabetes associated endothelial dysfunction and may cause premature atherosclerosis (Iwasaki et al., 2008). TNF-α and hyperglycemia have been shown to induce plasminogen activator inhibitor-1 (PAI-1) and cell adhesion molecules (VCAM-1 and ICAM-1) expression in human vascular endothelial cells (Morigi et al., 1998). Treatment with GLP-1-R agonists reduces TNF-α-mediated expression of PAI-1, ICAM-1, and VCAM-1 expression as well as TNF-α induced oxidative stress in human vascular endothelial cells (Liu et al., 2009; Shiraki et al., 2012).

Recent experimental studies have shown that GLP-1R activation by administration of GLP-1 analog exendin-4 significantly reduces accumulation of monocytes/macrophages in the vascular wall of C57BL/6 and ApoE^−*/*−^ mice. This effect seems to be mediated by suppression of the inflammatory response in macrophages through the activation of cAMP/PKA pathway, which inhibits the expression of TNF-α and of monocyte chemoattractant protein-1 (MCP-1), two molecules involved in macrophage recruitment, an important event in early atherosclerogenesis (Arakawa et al., 2010).

The relevance of GLP-1 is further supported by the observation that its receptor is expressed on podocytes, suggesting that GLP-1 may have a potential role in diabetic nephropathy. Up-regulation of DPP-4 expression in renal glomeruli occurs during inflammation (Stefanovic et al., 1993), and this phenomenon is associated with the development of diabetes-induced glomerulosclerosis. A further observation is that GLP-1Rs were down-regulated in renal glomeruli and tubules of diabetic rats and DPP-4 inhibition up-regulated such expression (Liu et al., 2012). Evidence from investigations with DPP-4 inhibitors in diabetic eNOS knockout mice (Alter et al., 2012), a model of diabetic nephropathy, suggests the potential of DPP-4 inhibitors to reduce albumin excretion. This reduction in albumin excretion is thought to reflect beneficial effects of DPP-4 inhibition on podocytes in a context where podocyte loss is one of the first events leading to proteinuria (Sharkovska, 2011).

### Incretin hormones and central nervous system

Glucagon like peptide 1 receptor stimulation has been associated with cytoprotection and reduced apoptosis in several tissue types. This trophic action was likely mediated by PKA and by phosphoinositide 3-kinase (PI3K) signaling. GLP-1R stimulation has been shown to suppress pro-apoptotic protein Bax and to stimulate anti-apoptotic protein Bcl-2 expression, thereby favorably modifying Bax/Bcl-2 ratio, supporting cell survival. Pancreas, brain, and heart have been shown to express the same GLP-1R type and it may be speculated that this cytoprotective effect may occur in these tissues (Drucker, 2003; Ban et al., 2010; Li et al., 2010).

Recent studies have further demonstrated neurotrophic and neuroprotective effects of GLP-1 and GLP-1 analogs on learning and cognition, including reduced beta-amyloid expression, improved synaptic plasticity, improved object recognition, improved spatial learning and memory (Perry et al., 2002; During et al., 2003).

Specific hypothalamic nuclei serve as control centers for appetite. The arcuate nucleus lies outside the blood-brain barrier and is the major target for peripheral hormones that regulate appetite, including GLP-1. This nucleus contains two distinct types of neurons, anorexigenic and orexigenic, Among Neuropeptide Y-receptors (NPY-R), NPYR1 and Y5-receptor activation appears to stimulate appetite, while NPYRY2- and Y4-receptor activation suppresses food intake via presynaptic inhibition of NPY release. GLP-1 has been shown to antagonize the orexigenic effects of NPY. Of note, Neuropeptide Y system is able to regulate bone activity through specific receptors expressed both on osteoblasts and osteoclasts (Khor and Baldock, 2012). GLP-1R is also expressed in neurons projecting both into and out of areas of the central nervous system, which are critical to energy balance regulation (Larhammar, 1996; Hahn et al., 1998; Inui, 1999; Chaudhri et al., 2006; Egan and Margolskee, 2008). Increased GLP-1 levels result into an anorexigenic effect with a significant appetite reduction for the subsequent meal (Furuse et al., 1997; Kim et al., 2011).

## Diabetes Mellitus and Bone

An association between diabetes mellitus and loss of bone mass due to osteoporosis (OP) has been initially described in the 40s (Albright and Reifenstein, 1948). This discovery has more recently received great attention since the presence of OP, in diabetic patients, can increase morbidity and mortality. In recent years, several studies were carried out in order to better understand the effects of DM on bone metabolism (Christensen and Svendnsen, 1999; Gunczler et al., 2001; Strotmeyer et al., 2004).

### Diabetes and bone mineral density

A low BMD has been reported in T1DM, while in T2DM BMD is similar to or even higher than in non-diabetic subjects (Isidro and Ruan, 2010). Nevertheless, experimental evidence clearly demonstrated an increased fracture risk in T1DM and in T2DM (Vestergaard, 2007), due to decreased bone mass, altered bone composition, increased risk of falling, and/or to other factors such as the pharmacological treatment employed. Of note, the increased fracture risk was reported only in established T2DM patients in comparison with subjects with impaired glucose tolerance (IGT), a condition that precedes disease onset. Such difference can be explained by the anabolic effects of insulin on bone that may have protected IGT individuals from fracture risk. In addition, during disease progression, disease-related complications, such as glycation of collagen in bone, can contribute to increased bone fragility with consequent major fracture risk despite normal or higher BMD (De Liefde et al., 2005).

In T1DM and, to a lesser extent in T2DM, fracture risk is increased for most skeletal sites, such as humerus, hip, or spine; in T2DM (Vestergaard et al., 2005).

Overall, pathophysiological mechanisms behind above-mentioned phenomena can be divided into: (a) mechanisms that decrease BMD or weaken bone structure; (b) mechanisms that increase the likelihood of falls and of other traumas (Vestergaard, 2007).

#### Mechanisms that decrease BMD or weaken bone structure

Mechanisms underlying alterations of bone metabolism and turnover in DM are still unclear and a number of potential explanations have been related to the type of diabetes.

Type 1 diabetes mellitus is characterized by an absolute endogenous insulin deficiency, and the finding of osteopenia and OP in these young patients, has led to hypothesis that insulin exerts an anabolic action on bone (Thrailkill et al., 2005); indeed, insufficient skeletal mineralization during puberty has been proposed as a mechanism that may explain lower BMD in T1DM. This may be due to changes in insulin secretion resulting in a state of low bone turnover with a considerable reduction of number and activity of osteoblasts (Goodman and Hori, 1984). It has also been proposed that autoimmune and inflammatory phenomena, that precede T1DM onset, may contribute to bone loss or to altered mineralization during puberty (Räkel et al., 2008). Moreover, it has been reported that insulin-like growth factor-1 (IGF-1) may play an important role in the onset of bone alteration in T1DM due to its anabolic effects in childhood as well as in adulthood, through a direct action on osteoblasts, which express insulin and IGF-1 receptors. Of note in T1DM, serum IGF-1 and IGFBP-3 (IGF-Binding Protein-3) levels are lower than in T2DM and in non-diabetic patients (Jehle et al., 1998).

Recent studies have reported low IGF-1 levels together with high IGFBP-1 and growth hormone (GH) levels in adolescent T1DM girls with a disease duration>5 years, compared with controls, especially in the case of poor metabolic control (Moyer-Mileur et al., 2008). In addition, high blood glucose levels and disease duration were associated with increased bone resorption parameters, while low IGF-1 levels could be considered as good predictors of bone strength. It is important to emphasize that similar bone abnormalities have been described also in young diabetic patients with a fairly good glycemic control, thus reinforcing the concept that bone tissue is deeply influenced by glycometabolic control (De Schepper et al., 1998).

The influence of gender on the relationship between T1DM and OP is controversial, with some studies showing an increased risk in males vs. females (Strotmeyer et al., 2006); the use of oral contraceptives and high estrogen levels in premenopausal women may offer a protection against bone loss associated to diabetes.

In contrast with T1DM, which is characterized by insulin deficiency, T2DM is characterized by a state of insulin resistance and high insulin levels, at least in the first phase of the disease natural history. This may have positive effects on bone, while established T2DM has a net negative effect on skeletal integrity (Schwartz, 2009). Moreover, in non-diabetic subjects, a low Body Mass Index (BMI) is associated with decreased BMD and with increased risk of OP and of fracture (Espallargues et al., 2001). In a meta-analysis Vestergaard (2007) showed that BMI is also an important predictor of BMD in T2DM, while overweight and obesity, which are frequent in T2DM, are believed to be protective factors for BMD (Wang et al., 2005). These findings led to the hypothesis that, in diabetes, not only BMD but also bone quality and its microstructure could be impaired.

#### Mechanisms that increase the likelihood of falls and of other traumas

An interesting prospective cohort study (Schwartz et al., 2002) performed in 9249 women aged ≥67 years analyzed the number of falls: a total of 629 (6.8%) women had diabetes, including 99 who used insulin. During an average follow-up of 7.2 years, 1640 women (18%) fell more than once a year. Diabetes was associated with an increased risk of falling for patients not treated with insulin. In the first 2 years of follow-up, women with diabetes were not more likely to fall than those without diabetes, but they had significantly more falls. Women with diabetes were more likely to have additional risk factors for falls such as poor balance, arthritis, cardiovascular disease, depression, poor vision, and use of medications for sleeplessness or anxiety. These results indicate that older women with diabetes have a substantially increased risk for falls and suggest that fall prevention efforts should be taken into consideration in the treatment of older women with diabetes.

Another study (Bischoff et al., 2003) showed that a simple, inexpensive, and well-tolerated intervention such as vitamin D and calcium administration, may help in reducing the burden of falling in the elderly. Specifically, this is a double-blind randomized controlled trial, in which 122 elderly women (mean age 85.3 years) have been studied in long-stay geriatric care. Participants received 1200 mg calcium plus 800 IU cholecalciferol or 1200 mg calcium per day over a 12-week treatment period. The number of falls per person was compared between the treatment groups. The results showed that vitamin D and calcium supplementation reduced the number of falls per person by 49%, improved musculoskeletal function, increased vitamin D status, and decreased PTH secretion and bone resorption within 3 months of treatment.

### Adipokines, diabetes mellitus, and bone

The increase in visceral fat tissue, observed in T2DM, is usually associated to ongoing inflammation, which may contribute to an increased bone turnover, likely secondary to the release of adipokines by adipocytes (Baynes et al., 1997; Gysemans et al., 2002; Schaffler et al., 2006; Reid, 2008). A specific role in bone metabolism is played by leptin and adiponectin. In T2DM, circulating leptin levels are increased and are able to stimulate osteoblasts and to inhibit osteoclast formation and activity, thus promoting osteogenesis. In contrast, leptin seems to have an indirect negative effect on bone formation at the level of the central nervous system, acting on specific hypothalamic neurons. Consequently, the overall effect of leptin on bone results from the combination of negative and positive effects (Cirmanová et al., 2008; Kanazawa et al., 2009). In order to gain insights into the relationship between fat mass and BMD, an interesting study (Thomas et al., 2001), performed in premenopausal and postmenopausal women as well as in men, correlated leptin, insulin, and estrogen levels with BMD at the total hip, mid-lateral spine, and mid-distal radius. Results showed a correlation between serum leptin levels and BMD in women but not in men. Leptin was also negatively associated with bone turnover, suggesting its protective role against bone resorption.

As far as adiponectin is concerned, its effect is uncertain and has been related to bone turnover markers and to BMD (Miazgowski et al., 2012; Starup-Linde, 2013).

It is known that acute and chronic hyperglycemia suppress the expression of genes associated with maturation in mouse osteoblastic diabetic models, while increasing the expression of genes such as PPAR, which stimulates the differentiation of mesenchymal stem cells in adipocytes (Williams et al., 1997; McCabe, 2007; Merlotti et al., 2010). Indeed, the results of recent experimental investigations have shown that, similarly to what occurs in other tissues, a state of chronic hyperglycemia is able to induce non-enzymatic glycosylation and transformation of various proteins in advanced glycosylation end products (AGE), especially of type 1 collagen (Botolin and McCabe, 2006). Therefore, hyperglycemia and high oxidative stress, frequently observed in diabetes, would lead to formation of cross-glycosylated links to collagen chains that constitute the bone matrix, leading to a deterioration of bone mineralization and thus impairing biomechanical properties of the skeleton. AGEs can also affect bone metabolism by inducing the expression of pro-inflammatory cytokines such as TNF-alpha that promote resorption, or by inhibiting osteoblastic activity and maturation (Katayama et al., 1996; Saito and Marumo, 2010). A case-control study, performed in T1DM patients and in age- and sex-matched healthy subjects analyzed bone histomorphometric and micro-TC data on iliac biopsies. Results revealed no significant difference between the two groups, indicating no effects on bone structure before manifested diabetic complications (Armas et al., 2012).

It is important to emphasize that recent evidence has shown that bone tissue can affect carbohydrate metabolism; in particular, it seems that bone proteins such as osteocalcin (OC) (the main protein secreted by osteoblasts) can modulate insulin secretion as well as adiponectin expression in adipocytes, suggesting that bone secreted molecules might influence insulin secretion and glucose tolerance *in vivo* (Lee et al., 2007).

It has been hypothesized that both microangiopathy and macroangiopathy may contribute to OP and to increased fracture risk. As a matter of fact, diabetes-related comorbidities such as diabetic retinopathy, peripheral neuropathy, and cerebral stroke or hypoglycemia may increase the risk of falling. The combination of poor bone quality and frequent falls would be expected to increase the risk of fractures independently of BMD (Janghorbani et al., 2007).

### Bone turnover and diabetes mellitus

Several observations evidenced a condition of low bone turnover and decreased bone formation both in T1DM and T2DM (Janghorbani et al., 2007). A cross-sectional study evaluated serum levels of bone markers in 78 patients affected by T2DM compared with 55 non-diabetic subjects. Results showed lower levels of bone resorption markers and of i-PTH in T2DM compared with healthy subjects, while no difference was detected in bone formation markers. The hypothesis suggested by authors was that the lower i-PTH levels induced a low bone turnover state. This condition may be responsible of higher risk of fractures. However, conflicting results have been reported about the association between bone metabolism and T2DM. This can be due to different disease duration, influence of other factors such as estrogen, testosterone, ethnic group, and also the different bone site analyzed (e.g., cortical vs. cancelous bone) (Reyes-García et al., 2013).

Recently, a great interest has been focused on a new protein involved in bone formation, sclerostin. This protein is a secreted Wnt antagonist, produced almost exclusively by osteocytes, that binds to the low-density lipoprotein receptor-related proteins 5 and 6 (LRP5 and LRP6), thus inhibiting the canonical Wnt/β-catenin signaling pathway and thus osteoblast activity (McCabe, 2007). Its biological importance is underlined by both experimental studies in knockout animals and by clinical observations in subjects with sclerosteosis and van Buchem disease, two genetic disorders with impaired sclerostin production and markedly increased bone mass (Moester et al., 2010). In a recent study we showed (Gennari et al., 2012) a marked increase in circulating sclerostin levels in T2DM patients, with mean sclerostin concentrations>2-fold higher than in age- and sex-matched controls; this increase could represent a possible cause of reduced bone formation and of impaired bone quality observed in T2DM. A similar reduction in bone formation was also observed in T1DM, although sclerostin levels were not different between T1DM patients and control subjects, suggesting that partially different mechanisms may be involved in the pathogenesis of skeletal fragility in T1DM and T2DM. These results have been further confirmed by Gaudio et al. (2012) showing that sclerostin levels are associated with inhibition of Wnt/β-catenin signaling and reduced bone turnover in T2DM. In contrast, Jastrzebski et al. (2013) did not confirm the correlation between bone formation and serum sclerostin; however, such study was performed in mice that, in addition, were not diabetic, therefore in a quite different setting compared to the previous two studies (Gaudio et al., 2012; Gennari et al., 2012) that were conducted in T2DM patients.

Several drugs used for diabetes treatment have an effect on bone metabolism, not only through their hypoglycemic effect. Thiazolidinediones (TZD) such as pioglitazone, a peroxisome proliferator-activated receptor-γ (PPAR-γ) agonist, have recognized negative effects on bone. PPAR-γ is expressed in bone marrow cells and regulates mesenchymal stem cells differentiation into adipocytes or into osteoblasts. *In vitro* studies have shown that TZD stimulate mesenchymal stem cells differentiation into adipocytes rather than into osteoblasts (Ali et al., 2005). In agreement with these preclinical observations, several clinical studies demonstrated that long-term therapy with TZD causes BMD reduction and a great risk of fractures, particularly in females (Loke et al., 2009).

The use of metformin or sulfonylureas seems to have a positive effect on bone, with a reduction in fracture risk (Wang et al., 2005). Sulfonylureas might have a beneficial effect through the enhancement of IGF-1 secretion; experimental studies with cultured cells showed that metformin stimulates the differentiation and mineralization of osteoblasts (Kanazawa et al., 2008).

As for insulin, its anabolic effect on bone metabolism is well established; however, its effects on skeleton remain somehow controversial. Some studies demonstrated that insulin therapy was associated with a high risk of vertebral fractures (Kanazawa et al., 2010); this may be due to the fact that, for example, T2DM patients on insulin therapy are likely to have a long disease duration and/or complications, or a higher risk of hypoglycemic events.

## Incretin Hormones and Bone Metabolism

Bone turnover is characterized by a dynamic process that requires energy expenditure derived from meal ingestion. In particular, during the night, the lack of available nutrients could increase bone resorption in order to maintain stable levels in circulating calcium. After food intake, instead, bone resorption becomes unnecessary and it is consequently inhibited. This event may be mediated by gastrointestinal hormones released after meal ingestion such as GLP-1 and GIP (Henriksen et al., 2009). In line with this hypothesis, the observation that parenteral feeding is associated with reduced bone mass suggests that a deficit in incretin hormones could have a certain role in bone turnover (Henriksen et al., 2003).

**Figure 2:**
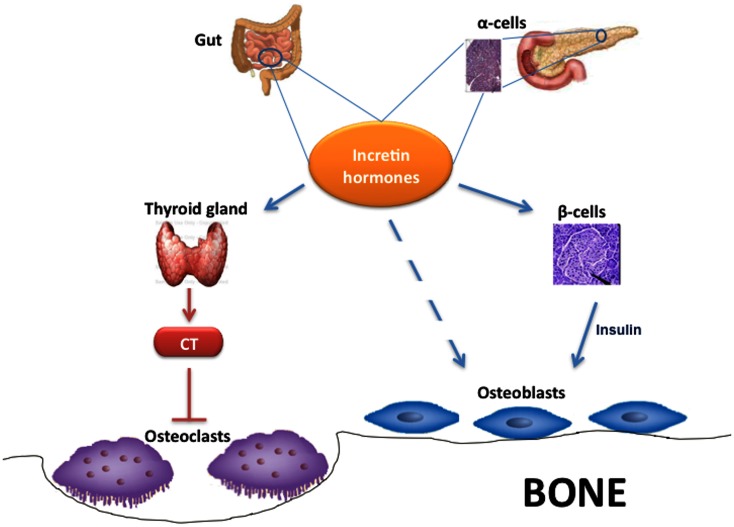
**Effects of incretin hormones on bone metabolism**. Incretin hormones are secreted by intestinal L-cells and, in minor amounts, by pancreatic α-cells. Incretin hormones can stimulate osteoblastogenesis indirectly via increased insulin secretion as well as through a direct action on osteoblasts. Moreover, incretin hormones can inhibit osteoclastogenesis by stimulating calcitonin production. CT, calcitonin.

**Figure 3:**
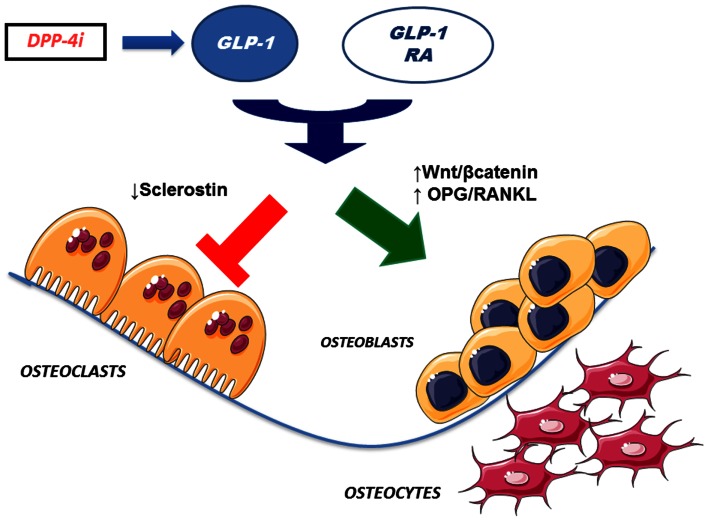
**Effects of incretin-based therapies on bone metabolism**. GLP-1 receptor agonists and DPP-4 inhibitors (via endogenous GLP-1) may stimulate osteoblastogenesis and inhibit osteoclastogenesis. Specifically, osteoblastogenesis stimulation has been hypothesized to occur via activation of Wnt/beta-catenin pathway and/or increased OPG/RANKL ratio. In addition, osteoclastogenesis inhibition has been suggested to be mediated by reduced sclerostin levels. GLP-1 RA, GLP-1 receptor agonists; DPP4i, dipeptidyl peptidase-4 inhibitors; OPG, osteoprotegerin; RANKL, receptor activator of nuclear factor kappa-B ligand.

Interestingly, some studies found that GLP-1 and other incretin hormones (Figure 2), such as GIP or GLP-2, could have a positive effects on bone through antiresorptive and anabolic properties, suggesting a beneficial effects of antidiabetic drugs like GLP-1R-agonists or DPP-4 inhibitors on bone metabolism (Figure 3, Table 1). However, the molecular mechanisms involved in such beneficial effects have not been elucidated and have only been hypothesized; these appear to involve Wnt/beta-catenin pathway, Osteoprotegerin (OPG)/RANKL (Receptor activator of nuclear factor kappa-B ligand) ratio and sclerostin levels.

**Table 1:** **Effects of incretin hormones on bone metabolism**.

Study subjects	Incretin hormones	Effects on bone	Reference
Postmenopausal women	GLP-2	↑BMD at cortical bone; ↓s-CTX; ↔s-calcium and s-phosphorous	Henriksen et al. (2009)
Human osteoblastic-like cells	GIP	↑Collagen type 1; ↑alkaline phosphatase, ↑osteoblast-like cell activity	Bollag et al. (2000)
Ovariectomized rats	GIP	↑BMD	Bollag et al. (2001)
Murine osteoclastic-like cells	GIP	↓Active bone resorption	Zhong et al., 2007
GLP-1R^−*/*−^mice	GLP-1	↓Cortical BMD	Yamada et al. (2008)
GPR^−*/*−^mice	GIP	↓Bone formation; ↑bone resorption; ↓bone mass	Xie et al. (2005)
GIP-overexpressing transgenic mice	GIP	↑Bone mass	Xie et al. (2007)
Murine C cell line	GLP-1	↑Calcitonin and bone resorption	Lamari et al. (1996)
Type 2 diabetic rats, insulin-resistant rats	GLP-1	↑OC; ↑OPG	Nuche-Berenguer et al. (2009)
Type 2 diabetic rats, insulin-resistant rats	Exendin-4	↑BMD	Nuche-Berenguer et al. (2010a)
MC3T3-EI osteoblastic cells	GLP-1	↑OC; ↔OPG	Nuche-Berenguer et al., 2010b
Hyperlipidemic rat models	GLP-1 or Exendin-4	↑OPG/RANKL ratio	Nuche-Berenguer et al. (2011)
Type 2 diabetic patients	Exenatide	↔BMD	Bunck et al. (2011)
Type 2 diabetic patients	Vildagliptin	↔BMD	Bunck et al. (2012)

*OC, osteocalcin; S-CTX, C-terminal telopeptide region of collagen type I; OPG: osteoprotegerin; BMD, bone mineral density; RANKL: receptor activator of nuclear factor kappa-B ligand*.

### GIP and bone metabolism

Bollag et al. investigated the expression of GIP-receptor (GIPR) in normal rat bone, in osteoblasts, and in osteocytes as well as in osteoblast-like cell lines, showing the presence of a high affinity, functional GIPR, that acts via cAMP pathway and increasing intracellular calcium levels. Moreover, GIP increases collagen type 1 expression and alkaline phosphatase activity in osteoblastic-like cells, consistent with an anabolic effect (Bollag et al., 2000) of this incretin hormone. These data have been confirmed in ovariectomized (OVX) rats compared with normal rats: intermittent injection of GIP in these models leads to a significant increase in lumbar BMD (BMD-L) only in OVX rats. Furthermore, they demonstrated *in vitro* that GIP action is dose- and time-dependent; indeed, high GIP concentrations down regulate GIPR expression in Saos-2 cells within 3 h of exposure; in contrast, lower GIP concentrations down regulate GIPR just after a longer exposure. Therefore, intermittent administration of GIP seems to have an anabolic effect and to prevent bone loss, similarly to PTH (Bollag et al., 2001). GIP receptors were detected also in murine osteoclastic-like cells, in which GIP appears to suppress RANKL-induced bone resorption (Zhong et al., 2007).

Using GIPR knockout mice (GIPR^−*/*−^), Xie et al. (2005) demonstrated a reduction in bone formation and an increase in bone resorption with a low bone mass; conversely, GIP-overexpressing transgenic mice exhibited increased bone mass (Xie et al., 2007).

### GLP-1, GLP-2, and bone metabolism

Additional investigations focused on the specific role of GLP-1 on bone metabolism. Yamada et al. (2008) demonstrated that GLP-1R^−*/*−^ mice are characterized by decreased cortical BMD, measured by quantitative computerized tomography (qTC), and by increased bone fragility. Moreover, bone histomorphometry showed that these genetically modified mice display increased osteoclastic bone resorption activity, which however appears not secondary to a direct action on osteoclasts. Furthermore, GLP-1 has also been shown to indirectly inhibit bone resorption via calcitonin; indeed, it has been reported that GLP-1R is expressed on thyroid C cells and that GLP-1 is able to stimulate calcitonin secretion (Lamari et al., 1996). Accordingly, calcitonin deficiency in GLP-1R^−*/*−^ mice could lead to increased osteoclastic bone resorption (Yamada et al., 2008).

In insulin resistant and in type 2 diabetic rats, the occurrence of an insulin- and PTH-independent bone anabolic action mediated by GLP-1 has been demonstrated, together with an osteogenic action on altered bone structure on osteoblasts. Overall, GLP-1 appears to have a double effect on bone metabolism, one direct and another indirect, the latter via thyroid C cells (Nuche-Berenguer et al., 2009). An experimental study was performed in animal models to analyze bone effects of GLP-1 administration in three different glycometabolic conditions: blood and bone samples were collected from streptozocin-induced diabetic, from fructose-induced insulin-resistant rat models, and from normal control rats, at basal and after 3 days of treatment with GLP-1 or placebo. This study showed an increase in OPG/RANKL ratio in both experimental models at bone level, but not in plasma. This difference between bone and plasma may derive from the acute administration and the short half-life of GLP-1. Nevertheless, a beneficial effect on bone structure was indeed observed in rats with impaired glucose metabolism (Nuche-Berenguer et al., 2010a).

A relationship between incretin hormones and bone has been investigated also in humans. Despite some differences with rodents, studies *in vitro* demonstrated that GLP-1 can functionally interact with human osteoblasts through a receptor different from GLP-1R described for pancreas (Nuche-Berenguer et al., 2010b). In osteoblastic MC3T3-EI cells, GLP-1 specifically binds to cell membrane and promotes the immediate hydrolysis of glycosylphosphatidylinositols (GPIs), thus generating diacylglycerol and inositol phosphoglycans (IPGs) that act as second messengers. GLP-1 induced phosphorylation in mitogen activated protein kinase (MAPK) through a cAMP-independent pathway, preferentially stimulated by GLP-1R coupled with G-protein expressed in other tissues. GLP-1 interaction with MC3T3-EI affected gene expression, in particular upregulated OC expression, while Wnt/β-catenin pathway remained unchanged (Nuche-Berenguer et al., 2010b). It was also described that osteoblastic-like cells, based on their stage of differentiation, differentially expressed receptors for gut hormones such as GIP, GLP-1, GLP-2, ghrelin, and obestatin. Expression of these molecules was assessed on three osteoblastic cells lines (Saos-2, TE-85, and MG-63) that represent different stages of osteoblastic development (Pacheco-Pantoja et al., 2011). Evidence of incretin effects in humans were provided by Henriksen et al. that showed a decrease in nocturnal bone resorption after administration of GLP-2 at 10 p.m. In this work, 160 postmenopausal women were randomized to receive daily doses of 0.4, 1.6, 3.2 mg of GLP-2 or placebo, plus calcium and vitamin D for 120 days. During this period women were monitored, reporting adverse effects and collecting blood and urine samples to assess safety and efficacy parameters. GLP-2 effects were evaluated together with circulating markers of bone formation and bone resorption and also BMD as measured by dual x-ray absorptiometry (DXA). Results reported the absence of any toxic effect of GLP-2 treatment and, more interestingly, a dose dependent increase in BMD, statistically significant for 1.6 and 3.2 mg doses compared to placebo. Injection of GLP-2 determined an immediate and sustained decrease in bone resorption markers such as C-terminal telopeptide region of collagen type I (s-CTX), while levels of bone formation markers, like OC, were not affected, thus providing evidence of a direct action of incretin hormones in regulation of bone metabolism (Henriksen et al., 2009). Subcutaneous injection of GLP-2 did not affect the same bone markers in colectomized patients with short-bowel syndrome evidencing the key role of small intestine in this response (Gottschalck et al., 2008).

### Exendin-4, exenatide, and bone metabolism

A similar approach was used in the same animal models employing GLP-1 analog exendin-4 (Ex-4). Also in this case, Ex-4 had an insulin-independent bone anabolic effect both in diabetic and in insulin-resistant rats, possibly due to the interaction with Wnt pathway (Nuche-Berenguer et al., 2010a). Finally, the same authors used GLP-1 and Ex-4 administration in hyperlipidemic rat models and observed similar osteogenic effects, thus confirming fat-bone relationships described previously (Nuche-Berenguer et al., 2011). A recent study by Kim et al. (2013) investigated cellular and molecular mechanisms responsible for the increased bone density following exendin-4 treatment, with specific focus on the effect of exendin-4 on osteocytes and on sclerostin production. First of all the presence of GLP-1 receptor was demonstrated, *in vitro*, on MLO-Y4 cells, a murine long bone-derived osteocytic cell line. Moreover, using immunohistochemistry and immunofluorescence, GLP-1R was shown to colocalize with sclerostin on osteocytes. Through quantitative real time RT-PCR and Western blot analysis, the authors demonstrated that exendin-4 treatment on MLO-Y4 cells reduced the production of sclerostin, both in normal than in high-glucose conditions. In addition, this study has tested the effects of exendin-4 treatment *in vivo* on Otsuka Long Evans Tokushima Fatty (OLETF) rats (a T2DM rat model) showing an increased femoral BMD together with a decrease of OC and unchanged levels of TRAP 5b, with a BMI independent effect. This suggests that a reduction of sclerostin in osteocytes and an increase of OC in osteoblasts might be related to a BMD increase (Kim et al., 2013).

Additional evidence of a positive effect of incretin hormones on bone turnover derived indirectly by data emerging from incretin-based therapy in T2DM patients. As reported above, T2DM is associated to an increased fracture risk (Vestergaard, 2007; Merlotti et al., 2010), and amelioration in bone parameters after incretin hormone administration has been reported. In a clinical trial, T2DM patients were randomized to receive GLP-1R-agonist exenatide or insulin glargine, added to their metformin-based therapy. The two groups were similar in terms of clinical characteristics (age, weight, sex, glycemic control, and markers of bone metabolism) at baseline. After 44 weeks of treatment, patients that received exenatide had a significant decrease in body weight compared to those treated with insulin glargine. The different drug regimen did not affect BMD, as shown by similar levels of BMD in the two groups at the end of follow-up. These findings suggest that, in T2DM patients, long-term injection of exenatide will not lead to an increase in fracture risk (Bunck et al., 2011).

### DPP-4 inhibitors and bone metabolism

A neutral role of DPP-4 inhibitors on bone metabolism was demonstrated by treatment with vildagliptin (100 mg once daily) in drug naïve type 2 diabetic patients for 1 year. Circulating levels of markers of bone resorption and calcium homeostasis were unaffected compared with baseline and to placebo (Bunck et al., 2012). A meta analysis was performed by Monami et al. (2011) on all trials that enrolled T2DM patients that received DPP-4 inhibitors for at least 24 weeks: fractures were reported only as adverse effects and probably not all types of fractures were described carefully; nevertheless, collected data revealed that the total number of fractures was lower in patients treated with DPP-4 inhibitors, suggesting a potential protective effect on bone of this class of drugs.

## Concluding Remarks

In conclusion, increasing evidence has shown that bone tissue is involved in pathophysiology of T2DM (Gaudio et al., 2012) as well as is a target organ of diabetic chronic complications.

In this complex scenario, several antidiabetic drugs have been demonstrated to influence bone metabolism. Among such drugs, the recently developed class of incretin-based therapy (GLP-1R-agonists and DPP-4 inhibitors), is of particular interest in the light of the effects of incretin hormones on bone metabolism. Specifically, data so far available both from experimental animal models and man, indicate that the beneficial “extra-glycemic” effects of this class of drugs include the effects on bone metabolism, that either directly on bone cells or indirectly (e.g., via thyroid C cells and calcitonin), appear to favor bone formation and to inhibit bone resorption, thus improving bone strength.

Further experimental and long-term clinical studies will be required to confirm the above-mentioned effects and to identify the underlying pathogenetic mechanisms. This could be particularly important not only for a better understanding of the causes of skeletal fragility in diabetes but also for its potential therapeutic implications.

## Conflict of Interest Statement

The authors declare that the research was conducted in the absence of any commercial or financial relationships that could be construed as a potential conflict of interest.
